# Exploring a Problem-Based Learning Approach in Pharmaceutics

**DOI:** 10.3390/pharmacy5030053

**Published:** 2017-09-20

**Authors:** Barbara McKenzie, Alyson Brown

**Affiliations:** School of Pharmacy and Life Sciences, Robert Gordon University, Aberdeen AB10 7GJ, Scotland, UK; alyson.brown@rgu.ac.uk

**Keywords:** problem-based learning, deep learning, pharmacy, pharmaceutics

## Abstract

Objective. The basis of this study was to explore the impact of the initiation of a Problem-Base Learning (PBL) approach within a second-year pharmaceutics degree on a Master of Pharmacy programme, introduced as a way of improving deep learning and to foster independent learning. Design. A semi-structured interview was used to seek feedback from the students, and feedback from staff was secured though a focus group. A thematic approach was used for the analysis, once data saturation had been reached. Exam pass-rate statistics were also analysed. Assessment. Five parent themes were identified from the student interviews: Module structure, Promoting lifelong learning, Integration and future practice, Outcomes and Student experience. The third year exam pass rate improved by 12% in the year following the introduction of PBL in second year. Conclusions. Various recommendations were proposed to further improve the module, based on the findings of this study. These include improving feedback and support through tutorials, reducing the volume of directed study, as well as highlighting the relevance of pharmaceutics to the pharmacy degree. A long-term review would be needed to assess the full implications of PBL teaching within this course.

## 1. Introduction

Medicine Design and Manufacture (MDM) is a second-year module at SCQF (Scottish Credit and Qualifications Framework) level 8 (in a 12-level scale) which runs over two semesters on the pharmacy Master’s degree program. The module covers pharmaceutics, that is, the drug journey from raw ingredient to formulated drug-delivery system, such as a tablet or an ointment.

Prior to academic session 2013/2014, the MDM coursework was delivered as a series of set experiments, in which the students worked through a coursework based around a specific dosage formulation, such as an emulsion or a suppository. Each small group of students had a designated academic staff member to guide them through the process. The students were instructed how to perform relevant calculations, research the pre-formulation of the medicine and then complete the manufacturing stage, with the work structured around clear learning outcomes. The coursework assessment (two written short-answer coursework tests) was based around the four liquid and five tableting practical lab sessions, and contributed 20% of the overall module mark. The remaining 80% was allocated to the written exam, which was based around the lecture material. 

For the 2013/2014 session, the coursework sessions were re-designed by the module team to follow a problem-based learning (PBL) approach using a set of mini-projects, which required students to explore a pharmaceutical formulation in a relevant problem. PBL is a teaching method used to develop skills such as team working, listening and self-directed learning [1]. The problem allows the use of research and reasoning in order to progress and complete the task in a contextualised way. The laboratory sessions were organised into groups of seven or eight students (130 students in total), who were each provided with a drug name, and a designated academic supervisor who was instructed to facilitate the work of the students. The coursework sessions ran as a series of six labs in semester one and dealt with liquid dosage forms, whilst semester two dealt with solid dosage forms, and ran as eight labs. Each lab was scheduled for three hours, with the designated staff member available throughout. These topics were complemented by parallel lecture sessions [2]. This format reflects other institutions, such as that described by Romero et al. [3]. Students undertaking the PBL sessions were provided with coursework sheets and questions on the pre-formulation and physicochemical aspects of their designated drugs. These served as a guide to the learning objectives that are essential to define the teaching and learning parameters for each module [4]. The coursework assessment for semester one was a formative report with feedback, and a summative report was submitted for semester two. The summative report contributed 50% towards the overall module grade, whilst the written exam after semester one provided the remaining 50% of the mark.

Initially, each group was assigned a drug at random, and students were expected to complete the coursework sheets on the physicochemical properties of this drug using appropriate literature sources. Successful completion of the coursework sheets was intended to provide the students with an information base to build on when deciding, with the guidance of the supervisor, on an appropriate formulation to be taken forward in the remaining laboratory sessions. After completing the appropriate COSHH (Control of Substances Hazardous to Health) and risk assessment (for physical risks) forms, each of the groups was able to create an appropriate drug-delivery system. Each supervisor mentored five groups over two sessions, and was able to work closely with each of the groups.

At this stage students were instructed to research their allocated drug. This was a change from the didactic approach students had experienced in their first year of study, and there was some initial uncertainty and reluctance. As this was the first year that the module had been taught this way, both staff and students were apprehensive. After an initial period of non-activity, the students appeared to behave in one of two ways; some realised that they had to organise themselves well and work together to gain the knowledge required to complete the project, whilst other students had a more negative reaction. The pressure of learning for themselves was a new experience for many of the students, and one which they did not appear to enjoy and reacted negatively to. Staff reassured students and helped build their confidence to make decisions and act on them. The students were encouraged to put effort into the formative report, in order to gain as much feedback as possible for the summative assessment. This formative report and the subsequent feedback received was essential in giving students the opportunity to improve and prepare for the summative report. Many of the students indicated that at first they did not see the point of the formative report, however the majority of students appreciated the value of the constructive feedback they received in helping them to complete the summative report.

The initial experience of PBL proved difficult and stressful for many of the students, who were not familiar with learning independently, and who were unsure of how to approach the formative report. Despite an end-of-year tutorial to give specific guidance on this aspect to students, many of the groups did not follow this guidance.

As the module delivery was novel, it was difficult to predict how the students would react to this new mode of teaching. There were also other issues to deal with, such as the School’s relocation to another campus within the city. Students were required to manage their group workload appropriately to complete tasks. Problem-based learning requires motivation and cooperation from the whole group to complete the task [5,6,7]. Without this, the group can quickly break down into sub-groups, where a minority do the majority of the work, and the others do little to organise themselves and manage their time. Initially, several of the students made little contribution to the group effort and had to be given a reminder that their cooperation and engagement was an essential requirement of this form of learning. This highlighted that some of the students lacked group work and engagement skills, with many having had minimal experience of this approach prior to entering their second year. To help overcome this problem, engagement became part of the assessment structure for the second semester, and greatly improved the students’ approach as a result. This was achieved by introducing an individual summary (30% weighting for the coursework assessment). The students were also exposed to scientific literature for the first time, which was daunting to begin with, however many students coped well and incorporated this information into their reports without prompting from staff. These are essential for progression through the degree program and into professional practice. Being able to work independently as well as part of a team are important skills for pharmacists, and also for lifelong learning [8,9,10,11]. 

**Figure 1:**
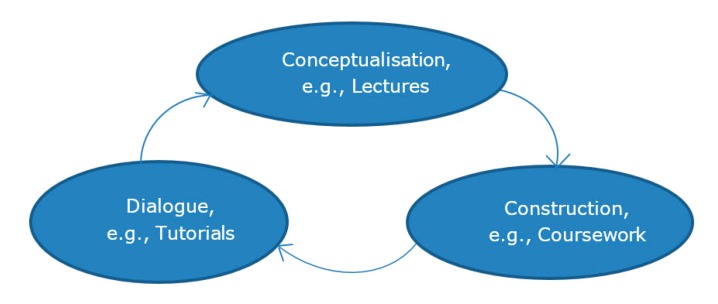
Mayes’ learning cycle.

When the students write their summative report for the coursework assessment, there is a requirement to link background theory to the lectures and directed study to the practical lab work, giving references and discussing the rationale behind their work [1]. This gives a context and a scientific basis for what they are trying to achieve. During semester two, all of the coursework related to tablet manufacture, so that all of the students were provided with the opportunity to go through the process. However, in semester one, each group chose a different type of liquid formulation and only manufactured one type of liquid. This meant that they could not build on the relevant lecture material in a practical way. This limits the deep learning which could occur, as the constructivism and connectivism mechanisms are restricted to theory only [12]. Many students need the practical aspect to fully understand and appreciate the information provided in lectures, and therefore these students could be disadvantaged. Mayes’ learning framework describes this in more detail (Figure 1) [13].

The teaching of background theory prior to practical lab experience involving PBL permits the assimilation of knowledge through reasoning and reflection [14]. This is the basis of Mayes’ learning framework [15], which deals with learning in a constructivist way. Constructivist learning is often problem-based, and allows students to build their own knowledge through supported tasks. This is compared to instructivism, which is a more traditional method of material delivery and requires rote learning. The structure of the new MDM module also allows cyclical learning to take place, from conceptualisation, through construction and finally to application, which is frequently dialogue in education (Figure 1) [15].

Conceptualisation is the interaction of new information with a student’s existing knowledge. Construction is the process of building knowledge through practical tasks. Finally, application is the testing of this new knowledge in applied contexts. There is a general trend in pharmacy education in the US towards PBL, as outlined in the Accreditation Council for Pharmacy Education (ACPE) Standards, and in the 2004 revision of the Centre for the Advancement of Pharmaceutical Education (CAPE) Educational Outcomes [16]. There is a move away from the simple transmission of facts, towards critical thinking and problem solving [17].

**Figure 2:**
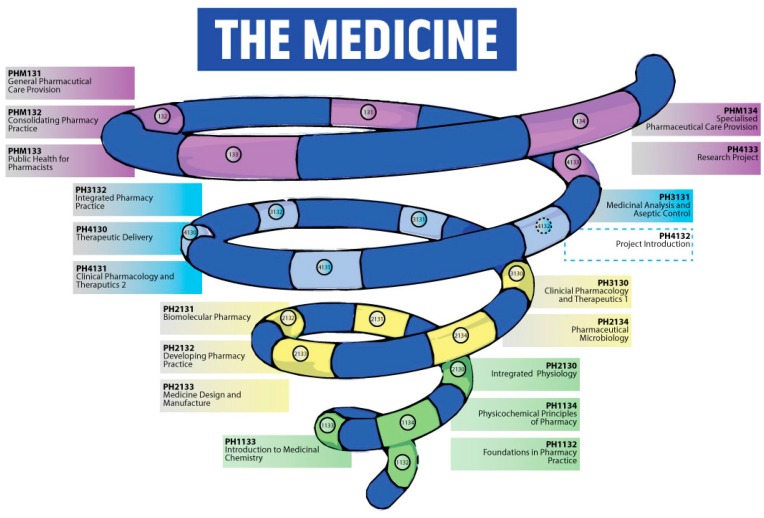
Schematic showing vertical learning through the MPharm degree course.

Conceptualisation begins when the students are introduced to new information during the lectures. This new knowledge is then built upon during the construction phase, where the students work through practical examples of the theory, and apply the theory to a practical context. This is now limited to the liquid which they choose to manufacture, however there is directed study which may help with this stage of learning. These two stages are then consolidated in the identification phase, which links the theory and relates it directly to the practical [18]. The current structure of the Master of Pharmacy (MPharm) degree is an upward spiral, allowing basic knowledge to be built on and reinforced, further promoting ‘deep learning’ (Figure 2). The old method of working through set coursework covered all of the lecture material in a practical way; however, this was not an ideal way to teach the subject. Certainly the feedback from students was that they preferred the new method of PBL compared to the more traditional approach which had an element of ‘spoon-feeding’, however, while they welcomed the PBL approach, they were of the view that it had gone from one extreme to the other, and would have preferred more input from staff. This was not helped by a lack of drug formulation in year one of the course. They also do not like that each staff member gave them a different answer to their questions, based on personal preference or experience. This is unfortunately undermining the students’ trust in the staff, and led to some major problems half way through the coursework. As this was the first year that MDM had been delivered in this way, there were always going to be teething problems, however, many of the students felt angry that they were ‘guinea pigs’ for this new method of module delivery. 

As a direct result of feedback received from the students, this year (2014/2015 session) the module was altered. Seminars and coursework covering some basic background and general techniques (didactic delivery of material) are carried out in semester one, before the summative work begins in semester two, which will be run as PBL. It is believed that this will improve the student experience, so that they are more prepared for the PBL, developing the relevant underpinning knowledge and improving the opportunity for deep learning with increased confidence. The basis of this study is to explore the impact of and reflect upon the 2014/2015 approach as compared to the 2013/2014 approach. It is the intention to seek feedback from the students at a later date on whether they consider their introduction to the PBL approach and associated independent learning helped them perform better in subsequent modules and in their third year of study. It is hoped that, by introducing PBL and a more independent learning style earlier in the degree, this form of teaching is made easier as they progress into other modules such as Therapeutic Delivery.

### 1.1. Design

This study explored student and staff views and attitudes on the introduction of PBL to the MDM module using a qualitative methodological approach. Semi-structured interviews were conducted with undergraduate pharmacy students followed by a focus group with staff involved in the delivery of the module. Ethical approval was obtained from the School of Pharmacy and Life Sciences Ethical Review Committee.

### 1.2. Recruitment

An invitation email was sent to all 2nd year (*n* = 130) and 3rd year (*n* = 128) undergraduate pharmacy students registered on the Master of Pharmacy program at the Robert Gordon University (RGU) in April 2015. An information leaflet (Appendix A) was attached to the email. A reminder email was sent to students after 2 weeks.

Pharmaceutics staff were invited by email to participate in a focus group. The information leaflet (Appendix A) was attached to the email, which was sent to all of the pharmaceutics staff (*n* = 6).

Informed consent was obtained from all participants prior to data being collected. Students were assured that their involvement would have no influence on their progression through the course.

### 1.3. Data Collection

Semi-structured interviews were conducted with students. Interviews lasted up to 20 min and were recorded on a digital voice recorder. An interview schedule was developed from existing literature and student feedback that had been gathered from existing module review processes, and reviewed by the research team before use. This was used as a guide for each interview and answers were explored further where appropriate.

A focus group was conducted with staff to validate themes that emerged from student interviews. The focus group topic guide was informed by the themes emerging from student interviews and the feedback gathered through the module review process.

### 1.4. Participants

Second-year students: Five second-year students volunteered to participate in the study (3.8%), and initially, five interviews were conducted. A further 2 interviews were then conducted to confirm data saturation.

Third-year students: Fifteen third-year students volunteered to participate in the study (11.7%), and initially, fifteen interviews were conducted. A further 2 interviews were then conducted to confirm data saturation.

### 1.5. Analysis

Each interview was transcribed verbatim following the conclusion of the interview, and analysis took place to identify emerging themes. Once data saturation was reached (i.e., no further themes were emerging), transcripts were independently analysed by another member of the research team to confirm no new themes were emerging and to validate the existing themes, and a further two interviews conducted to confirm saturation. The focus group transcript was analysed independently by two members of the research team and emerging themes validated [19,20,21].

### 1.6. Data Protection

All study materials were stored, processed and destroyed in accordance with Pharmacy and Life Sciences standard operating procedures. All data collected was stored securely and participants were assigned an anonymous code to ensure they could not be identified.

## 2. Evaluation and Assessment

**Figure 3:**
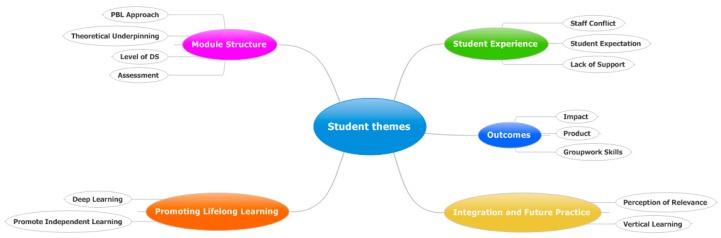
Mind map of the themes raised from the student interviews.

Five parent themes were identified from the student interviews: Module structure (17 comments in total), Promoting lifelong learning (7 comments in total), Integration and future practice (16 comments in total), Outcomes (12 comments in total) and Student experience (18 comments in total) (Figure 3).

Module structure is concerned with the use of PBL as a teaching method, how the module is assessed, the level of directed study (DS) and theoretical underpinning. Promoting lifelong learning is the overarching theme for deep learning and promoting independent learning. Integration and future practice involves the topics of vertical learning and the student’s perception of relevance. Outcomes are definable outputs such as group work skills, pharmaceutical product production and impact. Student experience involves student expectation, staff conflict and lack of support.

“Being able to apply your knowledge is the best way of learning.”

Participant 3.2F.

“It prepares you for the future… It’s what you need to do in reality.”

Participant 10.3F.

“There needs to be some sort of integration in terms of stepping up.”

Participant 3.3F.

“Our group made eye drops in first semester, but we didn’t learn about them until second semester… when it wasn’t really relevant anymore.”

Participant 2.3F.

“One DS had 40 questions… with no feedback.”

Participant 4.2F.

“That’s what employers look for, problem solving… you just need to get on with it, use your initiative.”

Participant 10.3F.

“Finding out the information yourself is sometimes more effective than someone just telling it all to you. You probably remember it better if you went and looked it up.”

Participant 2.3F.

“A lot of what I was reading and studying wasn’t relevant to be a pharmacist.”

Participant 5.2M.

“Vertical learning is starting to make sense now. I know that I have material from last year that I can look at.”

Participant 4.3F.

“It has improved my group work skills.”

Participant 4.2F.

“The best bit was finishing the tablet. I used my knowledge over 5–6 weeks, and made a tablet that could be used.”

Participant 3.2F.

“I felt like I knew a lot about suspensions at the end, but I didn’t really know a lot about the other formulations. Limited focus.”

Participant 8.3F.

“I felt I had no base knowledge.”

Participant 1.3F.

“I think it’s great, and it’s really useful to have the scope to be able to do things outside of constraints… it’s nice to be given the responsibility.”

Participant 7.2M.

“Misunderstanding between staff and students… that’s what caused the stress and frustration.”

Participant 3.3F.

**Figure 4:**
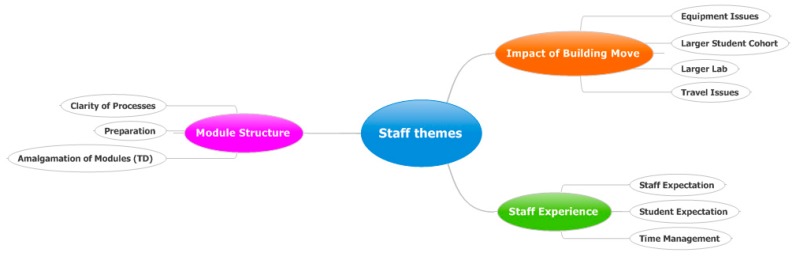
Mind map of the themes raised during the staff focus group.

Three overarching themes were identified from the staff focus group: Impact of building move (6 comments in total), Staff experience (7 comments in total) and Module structure (7 comments in total) (Figure 4).

Impact of building move encompasses issues such as the larger student cohort, larger lab, travel and equipment issues. Staff experience covers staff and student expectation and time management. Module structure involves staff preparation, module amalgamation (in the case of Therapeutic Delivery, TD) and clarity of processes.

“It was intensive, it required a lot of staff support.”

Participant 2SF.

“It was a perfect storm of a new, large lab, bigger cohort, a lot of walking, disorientation, not knowing where equipment is… I found that quite stressful.”

Participant 3SM.

“There was a lot of confusion amongst the students, because they are dealing with different individuals, with different experiences.”

Participant 3SM.

“Generally I don’t think students understand the concept that they are not being assessed on the quality of the products produced.”

Participant 2SF.

“It would be beneficial for us not to have 10 groups doing 10 different things. It’s exhausting.”

Participant 1SM.

“They’ve been exposed to the background, and because of it they are applying it and therefore they are more likely to remember it for next year.”

Participant 2SF.

## 3. Exam Results

**Figure 5:**
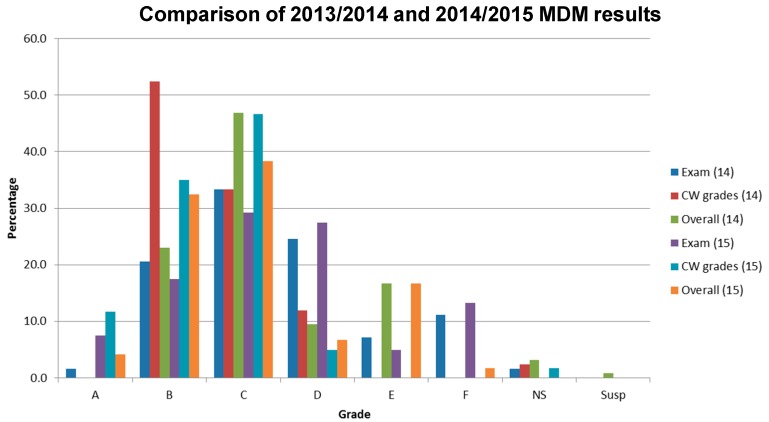
Comparison of Medicine Design and Manufacture (MDM) exam results between 2013/2014 and 2014/2015.

For the MDM module (2nd year), there was no significant difference between the results for 2015/2015 and 2013/2014. The assessment outcome was a pass rate of 82% in 2014/2015, compared to 79% in 2013/2014. The overall mean for 2013/2014 was 54% compared to 56% for 2014/2015 (Figure 5).

**Figure 6:**
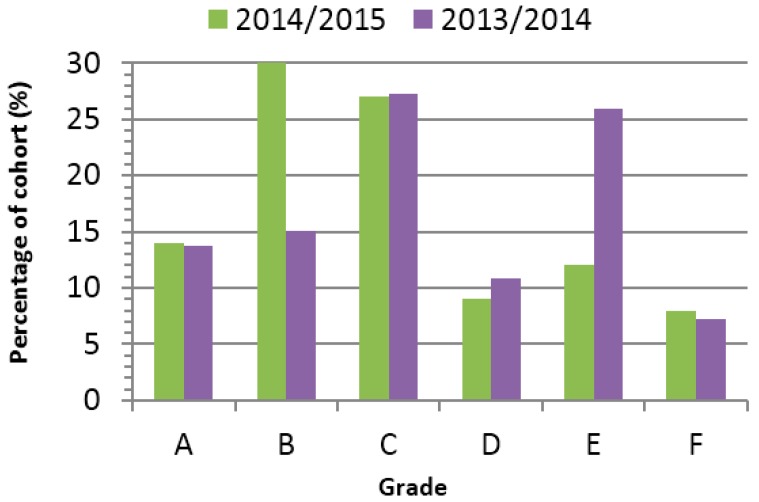
Comparison of Therapeutic Delivery (TD) exam results between 2013/2014 and 2014/2015.

The therapeutic delivery module (TD, 3rd year) enjoyed a significantly higher pass rate for session 2014/2015 compared to 2013/2014 (79% compared to 67%) (Figure 6).

## 4. Discussion

Key findings:

- Students enjoy the freedom of PBL, however, they need a lot of support to make this method successful.
- The level of DS needs to be reviewed.
- The relevance of the topics taught in MDM and TD needs to be emphasised to students.
- Communications between staff and students need to be improved, and outcomes clarified.

Overall, there were both positive and negative outcomes of the teaching year, and this is reflected in the positive and negative themes which emerged from interviews. The use of PBL in the new module structure was welcomed by the students. The 2013/2014 students enjoyed the freedom of the MDM module, and felt that having a structure where they were given the opportunity to put their skills and knowledge into practice benefitted their learning and their future practice; however, they also felt that they lacked support. This is not unusual for students undertaking problem-based or student-led learning for the first time. It is a different method of delivery and requires a lot of input from both staff and students. Giving the students this degree of responsibility so early in the course was a cause for concern during the planning stage, as some members of staff believed that the students were too inexperienced and used to being ‘spoon-fed’ to be able to deal with PBL effectively. However, after preparing the students during the first semester with set laboratory experiments, which covered all of the material they would be likely to encounter during semester two and was further backed up with tutorials and lectures, the students coped remarkably well. As a result of changes made to the module, the 2014/2015 students felt supported, although they felt that there could be more clarity in the expected outcomes. The level of DS came under scrutiny, as many students stated that they didn’t complete any of the DS, and nevertheless achieved over 60% in the exam. Many of the students completed the DS, but were unsure of the correct answers from a lack of feedback. With regard to the module exam, many students expressed concern that, out of eight questions, two were almost identical and one came from one lecture. They felt that it wasn’t representative of the module as a whole, nor of their future likelihood of dealing with the topics. The students also identified a lack of clarity regarding the module outcomes. Many students believed they were being assessed on the quality of the medicine that they made.

As part of the module reorganisation, the written exam was moved to the end of the first semester. This required that the students learn the material from semester one, before undertaking the practical project in semester two. It was believed that this would allow the ‘deep learning’ to fully occur. The third-year TD module enjoyed a significantly higher pass rate for 2014/2015 compared to 2013/2014 (79% compared to 67%) (Figure 6). There are many possible reasons for this. Firstly, the new amalgamated module had time to ‘bed in’, whereas last year was the first iteration of it. Staff were much more comfortable with the new lab layout, the larger student cohort and the projects they were running this year. This was also the first year of students who had been through the new PBL MDM module previously, so there is a possibility that the previous experience of a mini-project had prepared the students for the third-year module. These short-term improvements in pass rates have been seen previously in other studies [3].

For the MDM module, the assessment outcome was a pass rate of 82% for 2014/2015, compared to 79% for 2013/2014. The overall mean for 2013/2014 was 54% compared to 56% for 2014/2015 (Figure 5). There was no significant difference in short-term outcomes between the two academic years. When the MDM module was delivered as set coursework, before the 2013/2014 session, there were on average 20–30% of students who failed every year. For the 2013/2014 session, 18% of students failed. Further follow-up studies are required to determine if these affects are anomalies, and also if they have any impact on the third-year module TD.

Long-term outcomes are unknown at this point, and would require further study of postgraduates [22]. There is a possibility that PBL enhances students’ critical thinking and problem-solving skills, as well as their ability to learn independently.

Many of the students felt that they had experienced ‘deep learning’ due to the module’s structure. By making the students find out information for themselves, independent learning was also encouraged, which will be a vital career skill [10,11]. It is also a useful skill in many other areas of life. The results of this project are aligned with that of other studies that assess the outcome of PBL compared to a lecture-based module [22]. This study also reports that students feel significantly better-prepared for their future careers as a result of the PBL design, with regard to lifelong learning. There are many other published cases of successful PBL implementation [23].

Many of the students who were interviewed questioned the relevance of learning pharmaceutics in such detail, when they want to be career pharmacists. As the degree is at Master’s level, it incorporates surrounding elements of other relevant disciplines, such as human physiology, cell biology, sterile production and quantum mechanics. This is to provide a wide knowledge base, and allows many varied career options for our graduates. Having an underpinning knowledge of pharmaceutics allows our students to be medicine experts, and fully prepares them for whatever they might come across during their careers. Unfortunately, the implications of a strong background in science to underpin their knowledge as a pharmacist were not clear to them. There is therefore a clear need to further contextualise the module. Knowledge of the journey involved in making a medicine, from base chemical through manufacture to the patient administration, is of critical importance to the career of a pharmacist. The underpinning science of the MPharm degree serves not only to provide background knowledge—indeed, all pharmacists, whether they are working in industry, community, regulation or clinical settings will require in-depth knowledge of the area—but also the ability to utilise this knowledge on a daily basis. It is that which sets pharmacists aside from doctors, nurses and all other healthcare professionals; a deep and thorough understanding of all aspects of the medicines with which they work.

The vertical learning framework on which the MPharm course is based became apparent to the students (Figure 2). By introducing ideas slowly, and reinforcing them over the four-year course, ideas are built on and reinforced, becoming ‘deep’ knowledge.

Group work skills are important in the students’ future careers, as outlined in the General Pharmaceutical Council’s (GPhC) outcomes [10]. Being able to work effectively as part of a team is also attractive to future employers. During the first year of the MPharm degree course, there are very few group work exercises. Many students reported positive improvement in their group work skills during the second year. They also expressed pride over manufacturing their own dosage forms. Unfortunately, for those students who had a negative overall experience, the option of pharmaceutics as a final-year project was made untenable.

Key findings:

- There is a requirement for staff equipment training.
- The students need to be reminded of the relevance and importance of knowledge in pharmaceutics to their future careers.
- There is a need for improvement in the clarity of objectives and outcomes.
- The module structure in terms of learning outcomes and level of DS needs to be examined.
- The group sizes should be reduced in order to enhance lab experience.

There were a number of unique issues which came together to affect the overall student experience in the 2013/2014 semesters. In the new lab, 70 students could be taught at once, permitting a twice-a-week teaching schedule. As a result of this, more staff were required than originally thought. Students also discovered that staff have different areas of expertise, particularly with regard to equipment. This may have impacted on the students’ ability to work effectively in the lab sessions [23]. This feedback has identified a potential opportunity for staff training. In previous years it has been all too easy to ask for technical help from support staff during labs. In order to lessen that burden, staff training will be undertaken over the summer of 2015. There was also more pressure on staff, and some equipment was damaged during the move, impacting greatly on teaching. Despite highlighting the relevance of pharmaceutics as a module during the module introduction, students deemed the modules unnecessary and irrelevant. This produced a lack of engagement in some students, as well as disinterest and bad attitudes towards staff. Other science modules have reported the same problem, and therefore there is a need to emphasise the importance of a pharmaceutics background. Perhaps recently graduated students would be able to give an insight into how the science modules are useful in an everyday way for every pharmacist, whether they be working in community, hospital or industrial settings.

There was some confusion amongst students over outcomes of the module, particularly with regard to the coursework and what material was examinable. The amount of unsupported DS was a source of great frustration to the students, and was one of the most frequent topics raised by students during interviews. There needs to be improved communication generally between staff and students, and processes streamlined.

Due to the new lab size, many students and staff reported anxiety caused by too-large groups and too many people in the lab. Staff time had to be divided between the groups equally, which was challenging, and students felt there was not enough work for such large group sizes, which led to problems and resentment. This has been discussed at module review meetings, and will be reviewed for 2015/2016 semesters.

The implementation of PBL needs a lot of initial input from both staff and students, and so it was perhaps unsurprising that there were difficulties faced during the first year of the new module [24]. The first year of PBL implementation was a learning curve for both staff and students, and it is perhaps ill-advised to use it as a comparison. The second year of teaching the new module was much easier, and the students also had fewer criticisms, as the staff were also more comfortable with the new method of teaching. There is also the possibility that the previous year’s students gave feedback to the new cohort, easing anxieties and lessening unknowns. Only over time will we be able to monitor accurately for short-term outcomes [25].

Long-term outcomes are unknown at this point, and would require further study of postgraduates [25]. There is a possibility that PBL enhances students’ critical thinking and problem-solving skills, as well as their ability to learn independently. 

As a result of the student interviews and staff focus group, the following recommendations are suggested to address the issues raised by staff and students.

1. The relevance of the pharmaceutics aspect of the course must be highlighted to the students, in terms of their future careers. MDM is not the only module which the students deem ‘irrelevant’, and therefore a course-wide initiative is being planned with other staff from a science background.
2. The individual nature of each staff member’s experience and expertise can be utilised to create ‘equipment champions’, whereby each member of staff becomes a designated expert on certain pieces of equipment. This may help to eliminate frustration amongst students.
3. The volume of material included as directed study should be reviewed, and support such as feedback offered.
4. The exam set-up should be revised, and more multi-disciplinary questions included.
5. There should be an improvement in the clarity of objectives and outcomes made available to students.
6. Students should be introduced to all staff early in the module, and given an overview of their areas of expertise.
7. All staff involved in the MDM module are to be issued with coloured lab coats, to aid visibility during labs.
8. Student group sizes should be reduced, as many students are reporting having little to do.

To complete the cyclical process of review and reflection, further interviews and focus groups should be held to determine if any improvements have been made to overall module performance after the implementation of the above recommendations. Assessment results can also be compared from year to year, as a benchmark of learning.

## 5. Summary

Last year saw the introduction of a new remodelled PBL version of the MDM module. There were some issues raised by students at the time, and as a result of that feedback, improvements were made to the module. This is the second year that the module has been run, and the feedback was more positive. Staff are more comfortable with the module set-up, and there are less confounding issues. There are, however, still some issues which have come to light as a result of this project, and recommendations have been made to continue the process of continual module improvement. Despite a seemingly positive introduction to the MPharm degree, the results of this study are limited inasmuch as it only examines a short-term, cross-sectional outcome of the new MDM module, and it would require further, long-term reviews to determine the full impact of this method of module delivery.

## Appendix A

Dear potential participant

You are invited to take part in a research study. Thank you for taking the time to read the following information carefully. It is important for you to understand why the research is being done and what it will involve. Please ask us if there is anything that is not clear or if you would like more information. Take time to decide whether or not you wish to take part.

What is the purpose of the study?

As part of my Higher Education Learning and Teaching (HELT) course, I want to explore the student and staff experience of the MDM module, in particular comparing the experiences of the old module with the new, problem-based module.

Why have I been chosen?

All second and third year undergraduate students and pharmaceutics staff within the Faculty of Health and Social Care at Robert Gordon University will be invited to take part.

Do I have to take part?

No. Participation in the study is voluntary.

What will happen to me if I take part?

We ask that you complete a consent form, agreeing to take part in an interview. It will take place in March or April 2015 and you will be given a choice of date and time. Interviews will last around half an hour, and will be conducted on a one-to-one basis. Interviews will be recorded on a digital voice recorder.

What are the possible benefits of taking part?

There is no direct benefit to you from participation in this study. However, it is possible that findings will feed back into curricula development.

Will my contribution to this study be kept confidential?

All information gained in this project will be kept strictly confidential. Your name will not appear on any transcripts of the interview and all information will remain completely anonymous. Only the researcher will have access to the data collected.

What will happen to the results of the research study?

The results of the research study will be analysed and presented in an anonymised format in my final project and will be considered for publication.

Who is organising and funding the research?

This project is organised and funded by Robert Gordon University.

Who has reviewed the study?

This study has been approved by all Ethical Review Panels in the Faculty of Health and Social Care at Robert Gordon University.

To participate in this study, please complete consent form, available from McKenzie

Thank you! We’ll be in touch to agree the date and time for your interview.

McKenzie: b.mckenzie1@rgu.ac.uk; Tel: +44-01224-262588

## Appendix B

**Interview Questions**

1. Tell me about your experiences of the MDM module.
2. What were the best/worst aspects of the MDM module?
3. Can you describe the MDM exam?
4. How did the MDM module compare to other modules in the year?
5. How did the module Therapeutic Delivery compare to the MDM module? (Third year students only).
6. What are your thoughts on problem-based modules?
7. What impact do you feel the MDM module has had on your learning?
8. How would you improve the DMD module if were given the opportunity?
9. Is there anything else you would like to say about the MDM module?
